# Effect of silencing C-erbB-2 on esophageal carcinoma cell biological behaviors by inhibiting IGF-1 pathway activation

**DOI:** 10.1186/s13019-021-01540-9

**Published:** 2021-07-07

**Authors:** Zhigao Niu, Wenping Zhang, Jialun Shi, Xiangdong Li, Hanlei Wu

**Affiliations:** Cardiothoracic surgery department, Heping Hospital Affiliated to Changzhi Medical College, No. 110 Yan’an South Road, Luzhou District, Changzhi, 046000 Shanxi China

**Keywords:** C-erbB-2, IGF-1 signaling pathway, Esophageal carcinoma, Proliferation, Invasion, Migration, Cell cycle distribution, Apoptosis

## Abstract

**Objective:**

C-erbB-2 has been confirmed to be an oncogene that participates in cell growth, differentiation and division of tumors. We are wondered if its silenced expression can exert an anti-tumor effect. Therefore, this study is conducted to investigate the mechanism of C-erbB-2 silencing and IGF-1 pathway on esophageal carcinoma (EC) cell biological behaviors.

**Methods:**

The objects of study were 84 EC patients from Heping Hospital Affiliated to Changzhi Medical College, with the collection of EC tissue and adjacent normal tissue (> 5 cm away from cancer tissue). C-erbB-2 protein expression in EC tissues was detected by immunohistochemistry. Human EC cell line Eca-109 was purchased from Shanghai Institutes for Biological Sciences, Chinese Academy of Sciences. Based on different transfection protocols, EC cells with logarithmic growth phase of 3–5 passages were divided into blank control group, oe-C-erbB-2 NC group, siRNA C-erbB-2 NC group, oe-C-erbB-2 group, siRNA C-erbB-2 group, OSI-906 group, Rg5 group, Rg5 + siRNA C-erbB-2 NC group and Rg5 + siRNA C-erbB-2 group. Cell proliferation was detected by MTT assay; cell cycle distribution and apoptosis by flow cytometry; C-erbB-2, IGF-1, IGF-1R and Akt mRNA and protein expressions by qRT-PCR and western blot; and cell invasion and migration by Transwell assay and scratch test. Tumor growth was observed in male BALB/c nude mice (Shanghai Experimental Animal Center) based on Eca109 cell implantation, raising, and measurement.

**Results:**

C-erbB-2, IGF-1, IGF-1R and Akt expression were higher in EC tissues than those in adjacent tissues (all *P* < 0.05). Compared with blank control group, both si-C-erbB-2 and OSI-906 groups had decreased IGF-1, IGF-1R and Akt mRNA and protein expressions, decreased cell proliferation, migration and invasion, prolonged G0/G1 phase, shortened S phase, increased cell apoptosis, and inhibited tumor growth (all *P* < 0.05); while opposite trends were detected in C-erbB-2 vector and Rg5 groups (all *P* < 0.05), without statistical differences in siRNA C-erbB-2 + Rg5 group (all *P* > 0.05).

**Conclusion:**

Silencing C-erbB-2 expression may inhibit EC cell proliferation, promote cell apoptosis and block cell cycle progression by inhibiting IGF-1 pathway activation. The beneficial effect of silencing C-erbB-2 expression can be reversed by promoting the activation of IGF-1 pathway. Findings in our study may provide potential reference for understanding the molecular mechanism of EC and supply possible axis for preventing the development of EC from the perspective of molecular biology.

## Introduction

Esophageal carcinoma (EC) is the eighth most common cancer in the world, and its mortality rate ranks the sixth globally [1]. The incidence of EC has been increasing over the past decades [2]. The reason is not entirely clear, but it may be related to increasing risk factors such as smoking, drinking and obesity [3]. Esophageal squamous cell carcinoma is a major histological type of EC worldwide, most of which occur in Asia and Africa [4]. Endoscopic resection, surgical resection, chemotherapy and radiotherapy have been fully developed for the treatment of EC [5]. However, the treatment outcome of EC is far from satisfactory, and the 5-year survival rate is about 15–25% [6]. Due to the lack of early obvious symptoms of EC and the lack of effective early screening methods, most patients have been in the middle and late stage at the time of diagnosis and lost the best treatment opportunity [7]. Hence, the search for effective biomarkers of EC is of great significance in early screening, diagnosis and prognosis.

Insulin like growth factor 1 (IGF-1) and its receptor (IGF-1R) promote cell proliferation and inhibit apoptosis [8]. The role of IGF-1 in the development of tumor is a hot spot in current research. The biological function of IGF-1 is mediated by its specific target cell receptor (IGF-1R) [9]. IGF-1 signaling pathway plays an important role in promoting cell proliferation and inhibiting apoptosis [10]. Recent studies have shown that IGF-1 signaling pathway plays a key role in the development of lung cancer, breast cancer and colorectal cancer. IGF-1R can activate MAPK and P13K/Akt signaling pathways [11–13]. Recently, it has been found that IGF-1 signaling pathway may play an important role in various cancer types [11–14], and the key proteins in IGF-1 signaling pathway may become an effective point in the treatment of human malignancies.

With the deepening of research concerning molecular biology, tumor biotherapy has become a quite promising treatment after surgery, traditional radiotherapy and chemotherapy, among which molecular targeted therapy and gene therapy have achieved good results. Molecular targeted therapy takes specific marker molecules in the process of tumor growth as the target, and interferes with the signal transduction pathway corresponding to the target through specific blocking agents or blocking methods, so as to achieve the purpose of inhibiting tumor growth and promoting tumor cell apoptosis [15–17]. Molecular targeted therapy has a good selectivity to molecules and cells, it can thus make up for the disadvantages of common chemotherapy, such as poor selectivity and large side effects. It can effectively reduce the damage of drugs to normal tissues, with less adverse reactions [18, 19]. Gene therapy is the use of genetic engineering technology, such as a piece of DNA or RNA into the target cells or removed from the cells, so as to correct or compensate for the disease caused by gene defects and abnormal gene expression [20, 21]. It includes suicide gene, intervention for oncogene and tumor suppressor gene, gene therapy for inhibiting tumor angiogenesis, oncolytic virotherapy, etc. [22, 23]. It also includes gene silencing, which introduces exogenous or endogenous dual-stranded RNA into corresponding tumor cells, causing the degradation of homologous mRNA and further inhibiting the expression of corresponding genes in tumor cells [24, 25].

Among them, RNA interference (RNAi) is the main way of gene silencing [26]. Through RNA interference technology, the expression of key molecules in some signaling pathways during the growth of tumor cells is reduced, which inhibits the growth of tumor, and provides a new direction for tumor gene therapy [26, 27]. At present, the gene target of gene silencing therapy for EC is still in the process of research. Selecting a key gene with high specificity becomes the key of gene silencing therapy for EC. C-erbB-2 has been confirmed to be an oncogene that participates in cell growth, differentiation and division of tumors [28]. Changes in C-erbB-2 structure can result in overexpression of C-erbB-2 protein to enhance mitosis. There are various reports concerning the involvement of C-erbB-2 in human malignancies [29–32]. Here in our study, it was hypothesized that C-erbB-2 may be related to the occurrence and development of EC, with corresponding action of mechanism to be verified.

On the basis of the above interpretation, and considering the important roles of C-erbB-2 in EC, our study was carried out to investigate the regulation mechanism of IGF-1 signaling pathway mediated by C-erbB-2 on proliferation, apoptosis and cell cycle change of EC cells.

## Materials and methods

### Tissue sampling

EC tissue and adjacent normal tissue (> 5 cm away from cancer tissue) were collected from 84 cases of EC patients from Heping Hospital Affiliated to Changzhi Medical College. The fresh pathological tissue samples of all patients were divided into two parts. One part was quickly frozen in liquid nitrogen until total RNA was extracted, during which protein denaturation was avoided. The other part was fixed with formalin, and paraffin-embedded sections were prepared for immunohistochemistry. All the 84 patients were pathologically confirmed (31–72 years old) with an average age of (48.55 ± 7.12) years old. In this study, the specimen collection and clinical data collection were approved by the ethics committee of the hospital (approval No.: 2020–045), and the informed consent of all patients and/or their families was obtained before specimen collection.

### Immunohistochemistry

The EC tissue was embedded in paraffin for the preparation of serial sections of 4 μm in thickness, followed by conventional ethanol gradient dehydration and paraffin embedding. C-erbB-2 positive cells were detected by streptavidin peroxidase method. At 64 °C for 1–2 h until the wax was melted, the slice was put into xylene I for 5 min and xylene II for 10 min. After transparent processing, the slice was dried with absorbent paper and then added 100% ethanol I, 100% ethanol II, 95% ethanol, 85% ethanol, 70% ethanol, mixed with 3% H_2_O_2_ and distilled water (1:10) for 5–10 min at room temperature to block the inactivation of endogenous peroxidase. Following PBS washing (3 × 5 min), slices were placed in 0.01 M citric acid buffer (pH 6.0), boiled (95 °C, 5–10 min), and cooled naturally for > 20 min. With repeated PBS washing, 5% bovine serum albumin (BSA) blocking solution was added to the slices and reacted for 20 min at room temperature. In the next step, an appropriate amount of diluted rabbit monoclonal antibody C-erbB-2 was added overnight at 4 °C, and biotinylated goat anti-mouse IgG HRP secondary antibody was added for incubation at 37 °C for 30 min. SABC was added and incubated at 37 °C for 20 min, followed by PBS washing (3 × 5 min). DAB development was performed at room temperature, and the reaction time was controlled by observing the development degree under the microscope, which was generally 5–30 min. After mild staining with hematoxylin, the slices were dehydrated, followed by transparent processing with xylene for 5 min, and sealing with neutral gum. The results were observed under the microscope, with the selection of 5 high power mirror fields (× 400) randomly. The total number of tumor cells and the number of positive cells were counted, and the percentage of positive cells was calculated. All results were independently read and interpreted by two pathologists.

### Cell transfection and grouping

Human EC cell line Eca-109 was purchased from Shanghai Institutes for Biological Sciences, Chinese Academy of Sciences. The cryopreserved tumor cells were removed from liquid nitrogen and placed in a heated water bath at 37 °C. The cryopreserved cells were dispersed by shaking the cryopreservation tube gently. A volume of 5 ml cell culture medium was added into each tube in advance. After the cells were completely dissolved, they were centrifuged at 1000 rpm for 5 min. After discarding the supernatant, 2 ml RPMI-1640 medium containing 10% FBS was added into each tube, blown gently and mixed well, then transferred into sterile culture flask, supplemented with culture medium until the culture medium covered the bottom of the flask, and the culture flask should be placed horizontally without overflow when moving. The culture flask was then placed in a 5% CO_2_ incubator at 37 °C. The cells can be passaged after reaching 70–80% fusion. In the next step, complete medium containing 10% FBS, twice the volume of trypsin, was added to terminate digestion. The cells were separated from the culture flask and evenly suspended into single cell suspension. After that, the cell suspension was collected with sterile centrifuge tube, and centrifuged at 1000 rpm for 2–3 min. The supernatant was then discarded and added with complete culture medium for the preparation of single cell suspension by re-blowing with a pipette. Cells were then placed in a 5% CO_2_ incubator at 37 °C to culture for the observation of cell growth. The culture medium was changed every 2–3 days.

After stable passage, the cells at a cell fusion of 80% ~ 90% were digested by 0.25% trypsin, collected by centrifugation tube, centrifuged at 1000 rpm for 3–5 min, and then added into the prepared cell cryopreservation solution (10% DMSO, 90% FBS) for cell suspension, and stored in the cryopreservation tube after sub-package. Cells were stored at 4 °C for 2 h, transferred to − 20 °C for 2 h, then transferred to − 80 °C overnight, and then stored in liquid nitrogen the next day for further use.

In the process of cell transfection, EC cells with logarithmic growth phase of 3–5 passages were put into the 6-well plate the night before. When 75% of the cells at the bottom of the 6-well plate fused, the cells were transfected according to the instruction of Lipofectamine 2000. To be specific, 100ul pure RPMI1640 medium and target plasmid were put into sterile large EP tube and mixed evenly. The RPMI 1640 medium without bovine serum and Lipofectamine 2000 were mixed in EP tube and placed at room temperature for 5 min. The prepared two liquids were mixed together and kept at room temperature for 20 min. After that, the medium in the 6-well plate was replaced by the medium without bovine serum, and the reagents in the mixed solution were added to the 6-well plate respectively. After 4-6 h incubation in 5% CO_2_ incubator at 37 °C, it was replaced by complete medium. Real-time PCR was used to detect the transfection efficiency after 24 h of transfection and total RNA extraction from cells in each group.

After 24–48 h of culture, the cells were divided into groups according to the transfection conditions, and the effects of each transfection scheme on EC lesions were explored respectively. At the same time, the internal reference group was set up to verify the success of transfection. The transfection schemes were as follows: (1) for exploring the effect of C-erbB-2 expression on cell proliferation, apoptosis and cell cycle change, the following groups were constructed, including blank control group (cells with no treatment), oe-C-erbB-2 NC group (cells with the transfection of oe-C-erbB-2 negative control [NC] plasmid), siRNA C-erbB-2 NC group (cells with the transfection of siRNA C-erbB-2 NC plasmid), oe-C-erbB-2 group (cells with the transfection of oe-C-erbB-2 plasmid to overexpress C-erbB-2), siRNA C-erbB-2 group (cells with the transfection of siRNA C-erbB-2 plasmid to silence C-erbB-2 expression); (2) for exploring the effect of IGF-1 signaling pathway on cell proliferation, apoptosis and cell cycle change, another two groups of OSI-906 group (IGF-1 signaling pathway inhibitor to inactivate this pathway) and Rg5 group (Ginsenoside Rg5, IGF-1 signaling pathway agonist to activate this pathway) were established; (3) besides, Rg5 + siRNA C-erbB-2 NC group and Rg5 + siRNA C-erbB-2 group (pathway activation treatment using Rg5 combined with siRNA C-erbB-2 transfection) were built to clarify the relationship of C-erbB-2 and IGF-1 signaling pathway in the progression of EC.

### MTT for the detection of cell proliferation

Parental cells, blank plasmid control cells and experimental cells in logarithmic phase were inoculated into 96-well plates (5 parallel wells for each group), and the cell density was adjusted to 1 × 10^4^/well. The 96-well plates were placed in the cell incubator with 5% CO_2_ at 37 °C for 24 h, 48 h, 72 h and 96 h, respectively. Four hours before the end of culture, 20 μl of MTT solution was added into each well for 4 h; the supernatant was discarded and 150 μl DMSO solution was added into each well; and the prepared solution was shaken in a horizontal shaker to mix well. OD values of each well were detected at 490 nm. Each experiment was repeated three times. The cell viability curve was drawn with time as abscissa and OD value as ordinate.

### Scratch test for the detection of cell migration

The transfected EC cells were inoculated in 6-well plates, and the transfection density was 2 × 10^5^ cell /mL. After the cells adhered to the wall and covered with 6-well plate, 1–2 fine marks were made in the middle of the bottom of each well with sterile pipette tip to create an experimental model for the scratch test of the cultured cells. The cells were washed with PBS for 3 ti mes, and 2 ml RPMI1640 medium was added into the culture well, with the setting of the control group. At the time of 0 h, 24 h and 48 h after scratching, the distance of cell migration was observed under inverted microscope and photographed. Compared with the number of cells crossing the scratch in the control group, the percentage was calculated to reflect the ability of cell migration. The experiment was repeated three times.

### Transwell assay for the detection of cell invasion

Matrigel was dissolved overnight at 4 °C two days in advance, and the Matrigel was diluted with serum-free medium at a ratio of 1:7. All operations involving Matrigel were carried out on ice. The Matrigel invasion chamber was placed in a sterile 24-well plate, and 20 μL Matrigel was added into the chamber to make the matrix glue evenly cover the chamber bottom. After 24 h of transfection, EC cells in each group were cultured in serum-free RPMI1640 medium to prepare single cell suspension and counted under microscope (concentration of 60–80 × 10^4^/ml). In the lower chamber of Transwell, 600 μL medium containing 10% fetal bovine serum was added, and cells were added to each well in the upper chamber. The cells were placed at 37 °C and cultured in 5% CO_2_ incubator for 24 h. After abandoning the upper chamber medium, the upper chamber was carefully removed and cells that did not penetrate the membrane was lightly rubbed with a wet cotton swab. PBS was used to wash the chamber for three times. The chamber was fixed with 4% paraformaldehyde for 30 min. Crystal violet staining was then performed for 20 min, followed by photography under an inverted microscope. Five visual fields were randomly selected and the number of cells penetrating into the stromal membrane was counted to reflect cell invasion in each group.

### Flow cytometry for the detection of cell apoptosis

The cells in logarithmic growth phase were digested with 0.25% trypsin to prepare cell suspension; the cells in each group were collected in 5 ml centrifuge tube, centrifuged at 1300rmp for 5 min, and the supernatant was discarded. The cells were washed with 4 °C pre-cooled D-Hanks solution, centrifuged at 1300rmp for 5 min, and the supernatant was discarded. The cells were fixed with 75% ethanol for 1 h, followed by cell centrifugation and removal of the supernatant. The above step of D-Hanks was repeated. After cell centrifugation and removal of the supernatant, according to the number of cells in the centrifuge tube, 600–1000 μl of cell staining solution was added to re-suspend cells to ensure that the passing rate of cells was 300–800 cell/s. Flow cytometry was used to record the red fluorescence at 488 nm to detect cell cycle.

Furthermore, cell apoptosis was detected by Annexin V-FITC/PI double staining. The cell treatment was the same as that described in the above. Cells in each group were inoculated with 6-well plates, 2 ml per well. When the fusion degree of cell growth reached 70–80%, the cells were digested by trypsin, and the cells in the supernatant were collected together. The cells were washed with PBS solution and centrifuged at 1300 rmp for 5 min, and repeated twice. The cell precipitate was washed with 200 μL 1 × binding buffer buffer and centrifuged at 1300 rmp for 3 min, followed by the addition of 200 μL 1 × binding buffer to re-suspend cells. After that, 10 μL Annexin V-FITC and 5 μL PI were added to mix well gently, followed by staining at room temperature in dark for 10 ~ 15 min. Finally, with the addition of 300 μL biding buffer, cell apoptosis was detected by flow cytometry at the wavelength of 488 nm.

### qRT-PCR for the detection of mRNA expression

The operation was carried out according to the instructions of Trizol kit. When the cells grew and fused to about 80% density, cell centrifugation was performed at the speed of 2000 rpm for 5 min, 1 ml Trizol was added into the cells after the removal of supernatant, which were then blown and mixed with a pipette tip, placed at room temperature for 5 min, and transferred to 1.5 ml EP tube. A volume of 0.2 ml chloroform was added into each tube, shaken up and down violently for about 15 s; the mixture was then placed at room temperature for 10 min; and centrifuged at 12,800 rpm at 4 °C for 15 min. After centrifugation, the colorless liquid on the top layer was piped into a new 1.5 ml EP tube, and the same volume of isopropanol was added, and then it was left for 10 min–30 min at 4 °C. The next step was centrifugation at 12,000 rpm at 4 °C for 12 min, with the blowing of the supernatant gently. The cell mixture was added into 1 ml of 75% ethanol prepared by DEPC water, shaken by vortex shaker for 30 s, and centrifuged for 12 min at 12000 rpm at 4 °C, with the supernatant discarded. When the RNA precipitation was nearly transparent, appropriate amount of RNase-free solution was added to fully dissolve the precipitation, followed by the detection of the RNA concentration and quality. The concentration of RNA and the absorbance of A260/A280 were detected by Nanodrop 2000/2000c spectrophotometer. When A260/A280 was between 1.8 and 2.0, the purity of RNA met the requirements. The quality of RNA was detected by agarose gel electrophoresis. Complementary DNA (cDNA) was synthesized by reverse transcription using RNA as template, and the operation was carried out according to the instructions of Promega M-MLV kit. A volume of 0.5 μg/ul of reverse transcription primer (2ul), 2.0 μg of total RNA and RNase-free H_2_O were added into the 25 ul system; the mixture was evenly mixed, and the water bath was at 70 °C for 10 min. The reverse transcriptase cDNA was obtained by soaking the above system in water at 42 °C for 1 h and then 70 °C for 10 min, and then stored at − 20 °C. PCR amplification conditions were: pre-denaturation at 95 °C for 10 min; denaturation at 95 °C for, 2 min; annealing at 60 °C for 30s; and extension at 72 °C for 30s, in a total of 40 cycles. The relative expression of target gene in each cell line was calculated. The primer sequences of qRT-PCR are shown in Table 1. (This method was also suitable for tissue experiment).

**Table 1:** The primer sequences of qRT-PCR

Genes	Sequences (5′-3′)
C-erbB-2	F: CCTCTGACGTCCATCATCTA
	R: ATCTTCTGCTGCCGTCGCTT
IGF-1	F: GGATTTCAAGCAGAACTGTGTTTT
	R: CCTGGACCTTGAATTTTTTCTTTTT
IGF-1R	F: CCAAAACTGAAGCCGAGAAG
	R: TGCACCTGTCGATATCGATG
Akt	F: GAGCTGTTCTTCCACCTGTC
	R: TAATGTGCCCGTCCTTGT
GAPDH	F: CTCAGACACCATGGGGAAGGTGA
	R: ATGATCTT2GAGGCTGTTGTCATA

*F* forward, *R* reverse

### Western blot for the detection of protein expression

The cells in logarithmic growth phase were washed with PBS solution pre cooled at 4 °C. The cell culture flask was placed on ice, the cells were scraped off with Cell Scraper, and transferred to a 10 ml centrifuge tube. The cell lysate containing PMSF was added into the centrifuge tube, which was placed on the ice for 30 min. During this period, the centrifuge tube was shaken to make the cells fully lysed, and then cells were transferred to 1.5 ml centrifuge tube for centrifugation (12,000 rpm) at 4 °C for 5 min. The supernatant after centrifugation was aspirated and the solution was stored at − 20 °C. The protein concentration was determined by Bradford method. The prepared gel was placed in the electrophoresis tank and added with an electrophoretic buffer for pre-electrophoresis at 10 to 20 V voltage for 20 to 30 min to remove impurities in the gel. The Marker and samples were added to each lane (5 μl for each lane). After switching on the electrophoresis apparatus, electrophoresis was carried out at the constant voltage of 60 V for 30 min. When the Marker reached the separation gel, the voltage was changed to 90 V for 90 min of electrophoresis. After electrophoresis, the PVDF membrane was immersed in methanol solution for 30 min, and the membrane was transferred by wet transfer. After that, PVDF membrane was removed, TBTS solution was used for 3 min of washing, and the membrane was placed in 5% skimmed milk powder solution on a shaker with a speed of 50 rpm for 1 h of sealing at room temperature. In the step of primary antibody incubation, the PVDF membrane was laid flat in the self sealing bag, and diluted antibody was added to ensure that the PVDF membrane was in full contact with the antibody for overnight incubation at 4 °C. In the process of secondary antibody incubation, after taking out the membrane, the primary antibody was recovered, gently washed with TBST buffer solution for three times, the secondary antibody was added to ensure that the PVDF membrane was completely covered, followed by incubation for 60 min in a shaking table of 70 rpm and repeated washing with TBST buffer solution for three times. The same amount of A and B solution in the ECL fluorescence detection kit were taken and mixed in dark, dropped onto the membrane and put in the gel imager. The images were taken by Bio-Rad image analysis system (Bio-Rad, USA) and analyzed by Quantity One v4.6.2 software. The relative protein content was expressed by the gray value of target protein bands/that of β-actin protein bands. The experiment was repeated three times.

### Establishment of orthotopic tumor model and grouping in nude mice

Forty-five 4-week-old male BALB/c nude mice (16-18 g) were purchased from Shanghai Experimental Animal Center, Chinese Academy of Sciences. All operations were in accordance with the relevant regulations of our institution. They were randomly divided into 9 groups with 5 rats in each group, including empty vector group, C-erbB-2 vector NC group, C-erbB-2 vector group, sh-C-erbB-2 NC group, sh-C-erbB-2 group, OSI-906 group, Rg5 group, Rg5 + sh-C-erbB-2 NC group, and Rg5 + sh-C-erbB-2 group.

Human EC Eca109 cells were cultured in RPMI-1640 medium containing 10% fetal bovine serum, 100 U/ml penicillin and 100μg/ml streptomycin, and cultured at 37 °C with 5% CO_2_. The cells were digested with 0.25% trypsin and passaged with the culture medium replaced every 1-2d. The cells in logarithmic growth phase were taken for the experiment. In the process of virus infection, cell suspension with cell density of 3–5 × 10^4^ cells/ml was prepared in complete medium and inoculated in the medium. Cell transfection was finished according to the virus volume. For the establishment and grouping of Eca109 cell implanted tumor model in nude mice, BALB/c nude mice were implanted with corresponding cells subcutaneously to form orthotopic tumor. The mice were injected subcutaneously into the subcutaneous part of the left and right scapula with a 1 ml syringe. Each mouse was inoculated with 100ul and raised in sterile environment. The mental state and tumorigenesis of nude mice were observed at all times. When the tumor length exceeded 4 mm, the measurement shall be started every 3 days. The volume of tumor was calculated according to = length×width^2^÷2. All animal experiments were conducted in accordance with the guidelines of Ethics Committee in our hospital.

### Statistical analysis

SPSS 21.0 statistical software (SPSS Inc., Chicago, USA) was used for data analysis. Each experiment was repeated three times. The measurement data was expressed by mean ± standard deviation. The comparison between cancer tissue and adjacent tissues was paired t-test, other comparison between groups used independent sample t test, and one-way analysis of variance was used for comparison among multiple groups. *P* < 0.05 meant that there was statistical difference during the comparison.

## Results

### C-erbB-2 expression in EC and paracancerous tissues

**Fig. 1:**
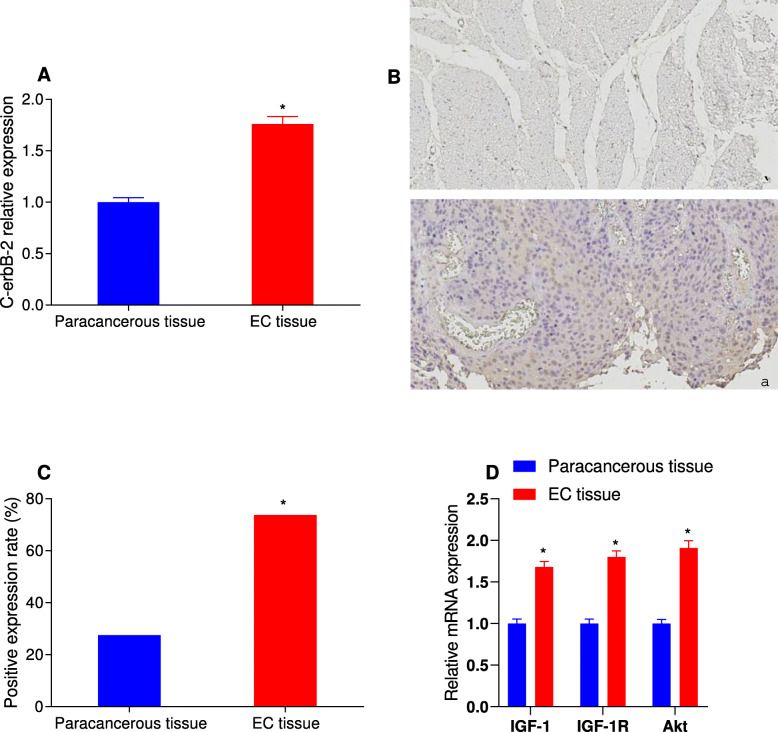
C-erbB-2 expression in EC and paracancerous tissues. Note: A. The expression of c-erbB-2 in EC tissues and paracancerous tissues detected by qRT-PCR; B: Immunohistochemical staining results of EC tissues and paracancerous tissues under high power microscope (× 400) (①paracancerous tissue; ② EC tissue); C: Positive expression rate of c-erbB-2 in EC tissues and paracancerous tissues detected by immunohistochemistry; D: The expression of IGF-1, IGF-1R and Akt in EC tissues and paracancerous tissues detected by qRT-PCR; *compared with paracancerous tissues, *P* < 0.05

C-erbB-2 expression in EC and matched paracancerous tissues were detected by real-time quantitative PCR. As shown in Fig. 1A, compared with the paracancerous tissues, the expression of C-erbB-2 was significantly increased in EC tissues (*P* < 0.05). The results of immunohistochemistry showed that the expression of C-erbB-2 was lower in the nucleus, mainly in the cytoplasm or cell membrane, which was stained in brown yellow; while there was no obvious staining in normal esophageal mucosa (Fig. 1B). Besides, 14 cases were positive and 36 cases were strong positive in EC tissues, and the positive rate was 62.50%. In paracancerous tissues, 61 cases were negative and 19 cases were positive, with the positive rate of 23.75%. The positive expression rate of C-erbB-2 in EC tissues was significantly higher than that in the paracancerous tissues (*P* < 0.05; Fig. 1C). At the same time, the mRNA expression levels of IGF-1, IGF-1R and Akt in EC tissues were significantly higher than those in the paracancerous tissues (*P* < 0.05; Fig. 1D).

### Effects of C-erbB-2 expression on cell proliferation, apoptosis and cell cycle

**Fig. 2:**
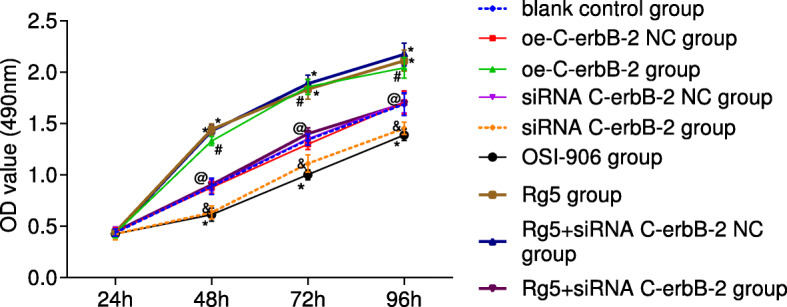
The proliferation ability changes in EC cells after cell transfection according to different grouping protocols in different groups. Note: *compared with blank control group, *P* < 0.05; #compared with oe-C-erbB-2 NC group, *P* < 0.05; &compared with siRNA C-erbB-2 NC group, *P* < 0.05; @compared with Rg5 + siRNA C-erbB-2 NC group, *P* < 0.05

Cells in blank control group, oe-C-erbB-2 NC group, oe-C-erbB-2 group, siRNA C-erbB-2 NC group and siRNA C-erbB-2 group were inoculated into 96-well plate. MTT method was used to observe the cell proliferation at 24 h, 48 h, 72 h and 96 h, and the relative proliferation rate was calculated. The results showed that there was no significant difference in cell proliferation at 24 h (*P* > 0.05). There were significant differences in cell proliferation rate at 48 h, 72 h and 96 h when compared with that at 24 h (all *P* < 0.05; Fig. 2). Compared with blank control group, there was no obvious difference in cell proliferation between oe-C-erbB-2 NC group and siRNA C-erbB-2 NC group (both *P* > 0.05); while oe-C-erbB-2 group had significantly accelerated cell proliferation than that in oe-C-erbB-2 NC group (*P* < 0.05), and siRNA C-erbB-2 group had retarded cell proliferation when compared with siRNA C-erbB-2 NC group (*P* < 0.05). These results indicate that up-regulation of C-erbB-2 can promote the growth and proliferation of EC cells, and down-regulation of C-erbB-2 can reverse this effect.

**Fig. 3:**
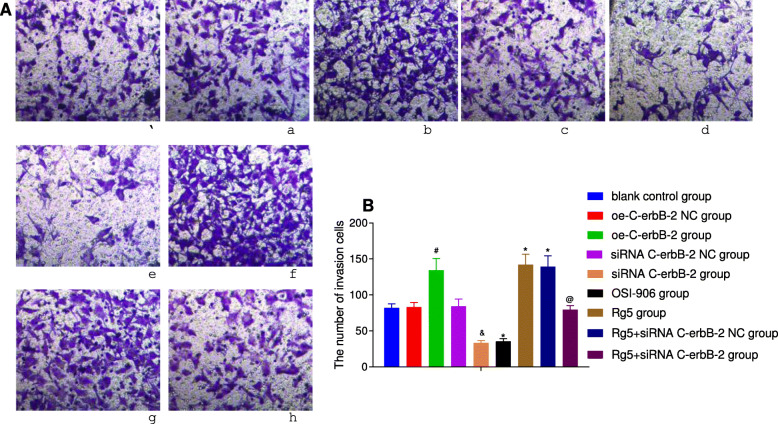
The invasion ability changes in EC cells after cell transfection according to different grouping protocols in different groups. Note: A: Cell invasion changes in different groups detected by transwell assay (①blank control group; ②oe-C-erbB-2 NC group;③oe-C-erbB-2 group; ④siRNA C-erbB-2 NC group; ⑤siRNA C-erbB-2 group; ⑥OSI-906 group; ⑦Rg5 group; ⑧Rg5 + siRNA C-erbB-2 NC group; ⑨Rg5 + siRNA C-erbB-2 group); B: Comparison of cell invasion in different groups; *compared with blank control group, *P* < 0.05; #compared with oe-C-erbB-2 NC group, *P* < 0.05; &compared with siRNA C-erbB-2 NC group, *P* < 0.05; @compared with Rg5 + siRNA C-erbB-2 NC group, *P* < 0.05

**Fig. 4:**
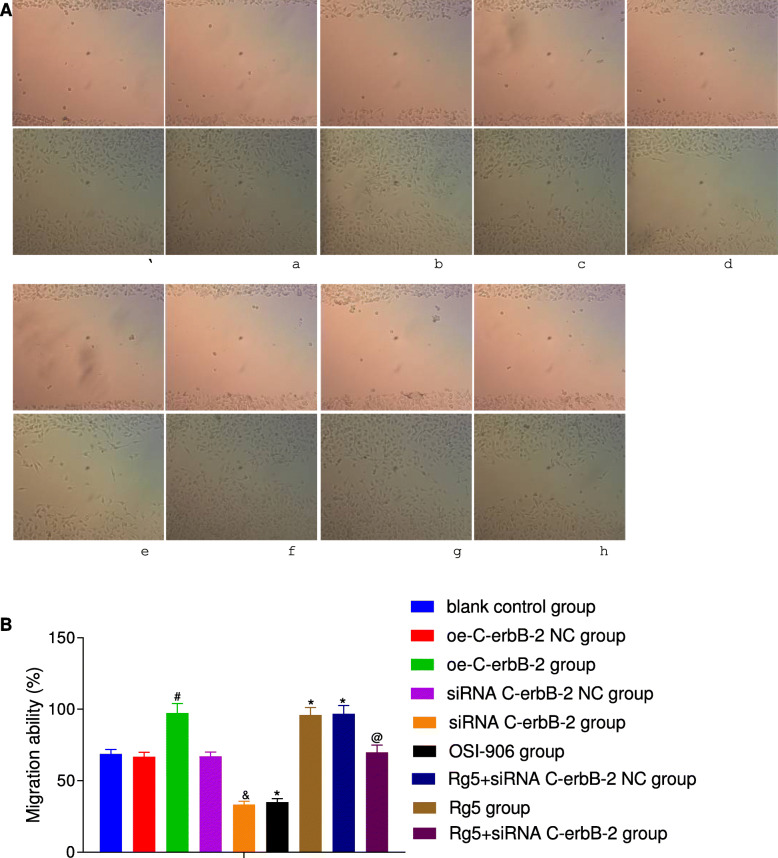
The migration ability changes in EC cells after cell transfection according to different grouping protocols in different groups. Note: A: Cell migration changes in different groups detected by scratch assay (①blank control group; ②oe-C-erbB-2 NC group; ③oe-C-erbB-2 group; ④siRNA C-erbB-2 NC group; ⑤siRNA C-erbB-2 group; ⑥OSI-906 group; ⑦Rg5 group; ⑧Rg5 + siRNA C-erbB-2 NC group; ⑨Rg5 + siRNA C-erbB-2 group); B: Comparison of cell migration in different groups; *compared with blank control group, *P* < 0.05; #compared with oe-C-erbB-2 NC group, *P* < 0.05; &compared with siRNA C-erbB-2 NC group, *P* < 0.05; @compared with Rg5 + siRNA C-erbB-2 NC group, *P* < 0.05

**Fig. 5:**
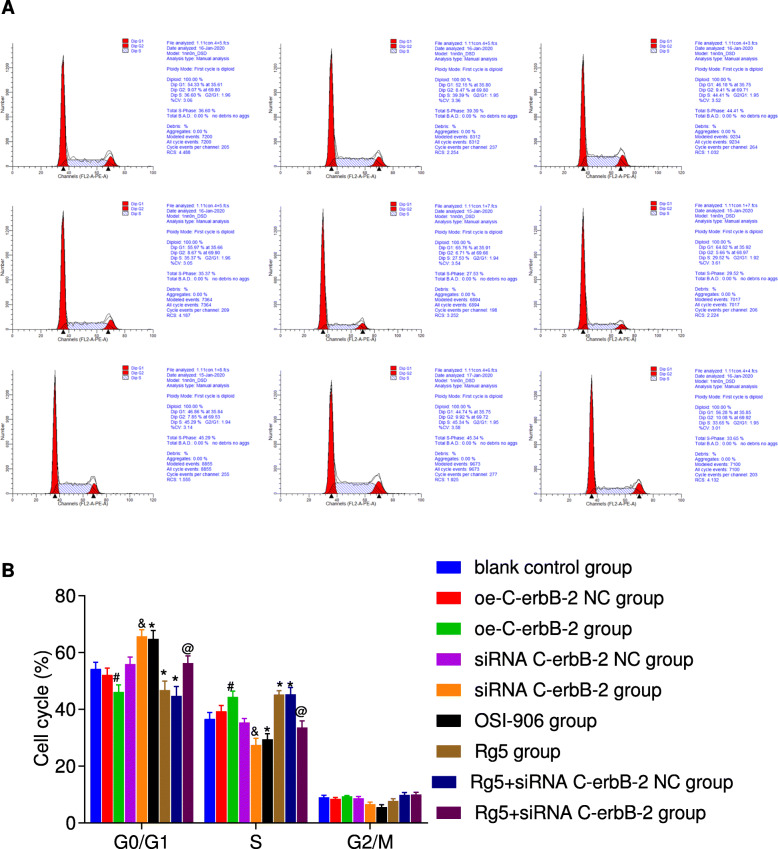
The cell cycle distribution changes in EC cells after cell transfection according to different grouping protocols in different groups. Note: A: Cell cycle distribution changes in different groups detected by flow cytometry (①blank control group; ②oe-C-erbB-2 NC group; ③oe-C-erbB-2 group; ④siRNA C-erbB-2 NC group; ⑤siRNA C-erbB-2 group; ⑥OSI-906 group; ⑦Rg5 group; ⑧Rg5 + siRNA C-erbB-2 NC group; ⑨Rg5 + siRNA C-erbB-2 group); B: Comparison of cell cycle distribution in different groups; *compared with blank control group, *P* < 0.05; #compared with oe-C-erbB-2 NC group, *P* < 0.05; &compared with siRNA C-erbB-2 NC group, *P* < 0.05; @compared with Rg5 + siRNA C-erbB-2 NC group, *P* < 0.05

**Fig. 6:**
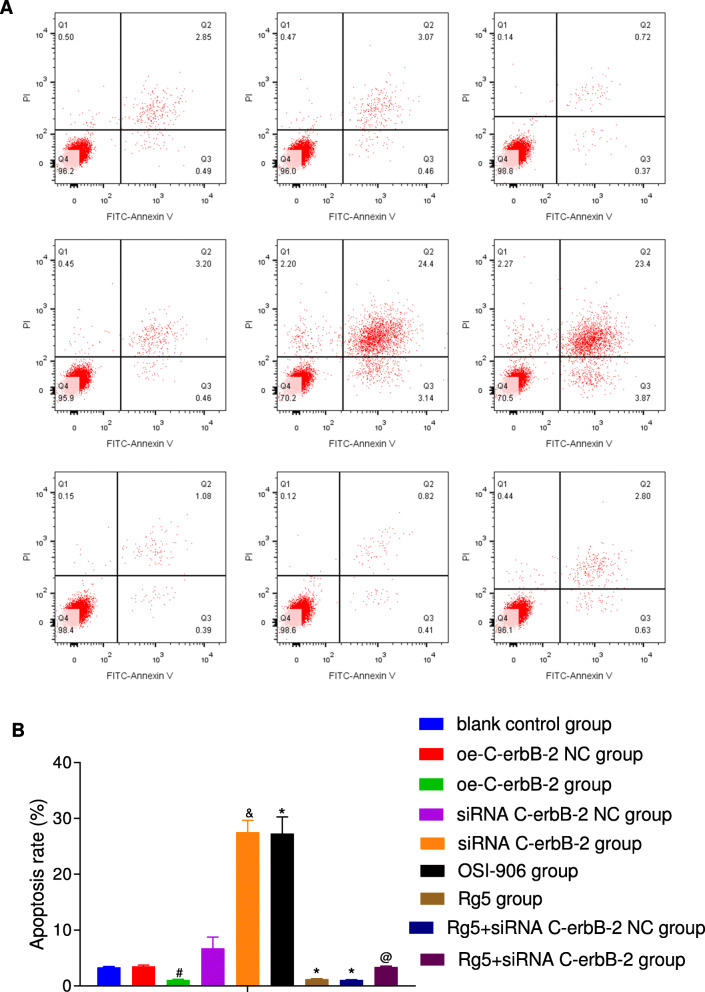
The cell apoptosis changes in EC cells after cell transfection according to different grouping protocols in different groups. Note: A: Cell apoptosis changes in different groups detected by Annexin V-FITC/PI double staining (①blank control group; ②oe-C-erbB-2 NC group; ③oe-C-erbB-2 group; ④siRNA C-erbB-2 NC group; ⑤siRNA C-erbB-2 group; ⑥OSI-906 group; ⑦Rg5 group; ⑧Rg5 + siRNA C-erbB-2 NC group; ⑨Rg5 + siRNA C-erbB-2 group); B: Comparison of cell apoptosis in different groups; *compared with blank control group, *P* < 0.05; #compared with oe-C-erbB-2 NC group, *P* < 0.05; &compared with siRNA C-erbB-2 NC group, *P* < 0.05; @compared with Rg5 + siRNA C-erbB-2 NC group, *P* < 0.05

At the same time, according to the results of Transwell assay (Fig. 3), scratch test (Fig. 4), flow cytometry (Fig. 5) and Annexin V/PI double staining (Fig. 6), the migration and invasion ability, cell cycle distribution and apoptosis level of oe-C-erbB-2 NC group and siRNA C-erbB-2 NC group were not significantly changed compared with blank control group (all *P* > 0.05). Compared with oe-C-erbB-2 NC group, oe-C-erbB-2 group had significantly increased migration and invasion ability of cells, shortened G0/G1 phase (decreased cell proportion), prolonged S phase (increased cell proportion), and decreased apoptosis rate (all *P* < 0.05). While siRNA C-erbB-2 group had obvious decreased migration and invasion ability of cells, and prolonged G0/G1 phase (increased cell proportion) and shortened S phase (decreased cell proportion), and increased apoptosis rate (all *P* < 0.05). These results suggest that up-regulation of C-erbB-2 can promote the invasion and migration of EC cells, increase cell proportion at S phase, and downregulate cell apoptosis; while the down-regulation of C-erbB-2 can inhibit cell invasion and migration, and promote apoptosis.

**Fig. 7:**
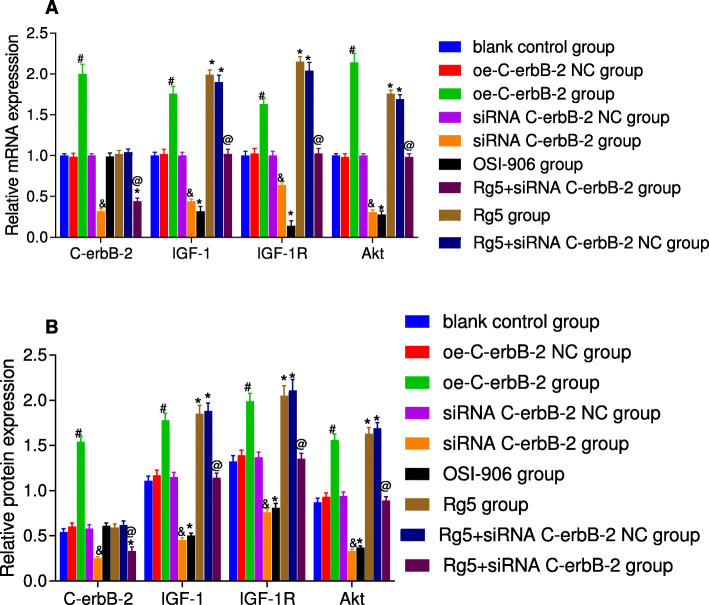
The mRNA and protein expressions of related indexes in EC cells after cell transfection according to different grouping protocols in different groups. Note: A: The relative mRNA expression in different groups; B: The relative protein expression in different groups; *compared with blank control group, *P* < 0.05; #compared with oe-C-erbB-2 NC group, *P* < 0.05; &compared with siRNA C-erbB-2 NC group, *P* < 0.05; @compared with Rg5 + siRNA C-erbB-2 NC group, *P* < 0.05

In addition, according to the results of qRT-PCR and Western blot (Fig. 7), compared with blank control group, oe-C-erbB-2 NC group and siRNA-C-erbB-2 NC group exhibited none obvious change in the mRNA and protein expressions of C-erbB-2, IGF-1, IGF-1R and Akt (all *P* > 0.05). In oe-C-erbB-2 group, the mRNA and protein expressions of C-erbB-2, IGF-1, IGF-1R and Akt were all upregulated significantly when compared with oe-C-erbB-2 NC group (all *P* < 0.05); while in relative to siRNA C-erbB-2 NC group, siRNA C-erbB-2 group had evidently downregulated mRNA and protein expressions of C-erbB-2, IGF-1, IGF-1R and Akt (all *P* < 0.05). These results suggest that silencing C-erbB-2 may inhibit the expression of IGF-1 signaling pathway related proteins.

### Effect of IGF-1 signaling pathway on cell proliferation, apoptosis and cell cycle

The cell proliferation was also compared in OSI-906 group and Rg5 group with blank control group based on the results of MTT assay at 24 h, 48 h, 72 h and 96 h. There was no obvious difference among groups at 24 h (*P* > 0.05). Compared with the rate at 24 h, the proliferation rate of cells at 48 h, 72 h and 96 h was significantly different (all *P* < 0.05; Fig. 2). Compared with blank control group, Rg5 group had obviously accelerated cell proliferation (*P* < 0.05), while that in OSI-906 group was slowed obviously (*P* < 0.05). These results indicate that IGF-1R signaling pathway agonists can promote the growth of EC cells, and IGF-1R pathway inhibitor can reverse this effect.

Compared with blank control group, Rg5 group showed evidently increased invasion and migration abilities of cells (Figs. 3-4), shortened G0/G1 phase (decreased cell proportion), prolonged S phase (increased cell proportion) (Fig. 5), and decreased apoptosis rate (Fig. 6) (all *P* < 0.05). While OSI-906 group had obvious decreased migration and invasion ability of cells, prolonged G0/G1 phase (increased cell proportion) and shortened S phase (decreased cell proportion), and increased apoptosis rate (all *P* < 0.05). Accordingly, the inhibition of IGF-1 pathway can weaken the invasion, migration and proliferation of EC cells, and promote apoptosis, while IGF-1R pathway agonists can reverse the ability of promoting EC cell invasion, migration, and inhibit apoptosis.

Meanwhile, as shown in Fig. 7, compared with blank control group, there was no obvious difference in the mRNA and protein expressions of C-erbB-2 in both Rg5 group and OSI-906 group (*P* > 0.05); while there were significantly increased trends in the mRNA and protein expressions of IGF-1, IGF-1R and Akt (all *P* < 0.05); besides, OSI-906 group showed remarkably decreased mRNA and protein expressions of IGF-1, IGF-1R and Akt (all *P* < 0.05).

### C-erbB-2 expression mediated the effect of IGF-1 signaling pathway on cell proliferation, apoptosis and cell cycle

Compared with blank control group, there was no significant change in cell proliferation (Fig. 2), invasion (Fig. 3), migration (Fig. 4), cell cycle distribution (Fig. 5) and apoptosis (Fig. 6) in Rg5 + siRNA C-erbB-2 group (all *P* > 0.05). However, compared with Rg5 + siRNA C-erbB-2 NC group, Rg5 + siRNA C-erbB-2 group had significantly accelerated cell proliferation rate, enhanced cell migration and invasion, shortened G0/G1 phase (decreased cell proportion), prolonged S phase (increased cell proportion), and reduced cell apoptosis (all *P* < 0.05). Meanwhile, Rg5 + siRNA C-erbB-2 group also exhibited significantly decreased mRNA and protein expressions of C-erbB-2, while obviously increased IGF-1, IGF-1R and Akt mRNA and protein expressions when compared with Rg5 + siRNA C-erbB-2 NC group (all *P* < 0.05; Fig. 7). These results suggest that supplemented treatment by using GF1 signaling pathway agonists can reverse the positive effect of C-erbB-2 silencing on cell proliferation, migration, invasion, cell cycle distribution and apoptosis.

### The volume and mass changes in tumor formation test and its metastasis in nude mice

**Fig. 8:**
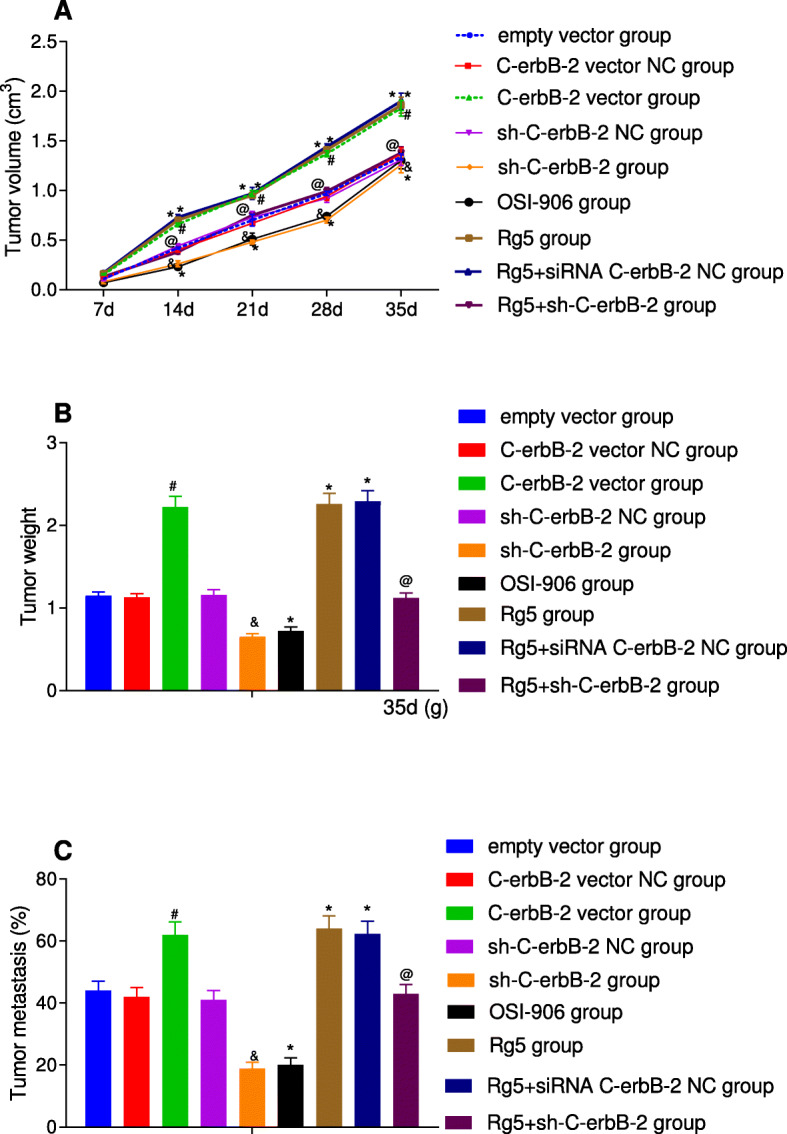
The change in the tumor volume, mass and metastasis of nude mice in each group after different interventions. Note: A: Tumor volume growth in different groups; B: Tumor mass size at 35d in different groups; C: Tumor metastasis rate in different groups; *compared with blank control group, *P* < 0.05; #compared with C-erbB-2 vector NC group, *P* < 0.05; &compared with sh-C-erbB-2 NC group, *P* < 0.05; @compared with Rg5 + sh-C-erbB-2 NC group, *P* < 0.05

The volume, mass and metastasis of transplanted tumor were measured in nude mice experiment and the results were shown in Fig. 8. Compared with empty vector group, there was no significant difference in tumor volume, mass and metastasis rate in C-erbB-2 vector NC group, sh-C-erbB-2 NC group and Rg5 + sh-C-erbB-2 group (all *P* > 0.05); C-erbB-2 vector group and Rg5 group had highly increased tumor volume, mass and tumor metastasis rate (all *P* < 0.05); sh-C-erbB-2 group and OSI-906 group showed the opposite results with significantly inhibited tumor volume, mass of tumor and rate of tumor metastasis (all *P* < 0.05). Moreover, compared with sh-C-erbB-2 group, Rg5 + sh-C-erbB-2 group had obviously increased tumor volume, mass of tumor and rate of tumor metastasis (all *P* < 0.05). These results suggest in animal model that overexpression of C-erbB-2 and activation of IGF-1 signaling pathway can promote the rapid growth and metastasis of tumor cells, while low expression of C-erbB-2 and the inhibition of IGF-1 signaling pathway can inhibit the growth and metastasis of EC cells.

## Discussion

EC is one of the malignant tumors with high mortality and destruction globally. It is a common malignant tumor of digestive tract with high invasiveness and metastasis [33]. Despite the continuous improvement of diagnostic criteria and treatment methods in the past few decades, high recurrence and mortality are still major obstacles to long-term survival of EC patients [34–36]. EC patients show none obvious symptoms in the early stage, and most of the patients are in the middle and late stage when diagnosed. Therefore, it is very important to identify the mechanism of EC as early as possible, and to find potential markers of early diagnosis and effective targets of treatment for EC patients.

Tumor invasion and metastasis are complex processes involving multiple factors, steps and stages [37]. Multiple genes, proteins and cytokines interact with each other to participate in and regulate the metastasis and dissemination of tumor cells from the primary site to adjacent tissues and distant organs [38–40]. Target therapy is an important progress in the field of cancer treatment research recently. Anti-tumor agents developed for specific molecular targets have achieved significant therapeutic effects in some cancer patients [40, 41]. RNAi is a process mediated by dual-stranded RNA that blocks the expression of corresponding gene sequences at the post-transcriptional level, which exhibits high efficiency in inhibiting gene expression [42]. It can realize an inhibition of the expression of target genes efficiently, specifically and rapidly. Therefore, it has been used in the research of tumor pathogenesis and corresponding treatment in recent years, with good results achieved.

In vitro experiment is an important method for basic research of tumors,. Despite the existence of differences in the environment in vitro and in vivo, tumor cell lines in vitro have most of the physiological and pathological characteristics of tumor cells in vivo, which can thus be applied to explore human tumors externally. It helps to obtain cells/tissues with uniform biological features that can be convenient for research at the molecular level. It can facilitate the observation of tumor cell growth, invasion and metastasis dynamically, and the understanding of genetic behaviors of tumors. More importantly, it can be helpful for rapid screening and research of anti-tumor agents, carcinogens and mutagens. In addition, Eca-109 EC cell line can grow rapidly, has high stability, and is easy to be cultured and passaged, which is one of the most commonly used cell lines for in vitro experiments of EC.

In our study, the expressions of C-erbB-2, IGF-1, IGF-1R and Akt mRNA in EC tissues were significantly higher than those in adjacent tissues. It provided our research team with insights that silencing C-erbB-2 or inactivating IGF-1 signaling pathway may exert protective role in the development of EC. More importantly, in view of the aforementioned understanding, our experiment used siRNA expression vector for in vitro cell test to identify the role of C-erbB-2 gene intervention. Corresponding plasmids were transfected into tumor cells by liposome transfection method, and satisfactory gene inhibition effect was obtained. According to the results, silencing the expression of C-erbB-2 resulted in the decreased expression of C-erbB-2, with no change after inactivating IGF-1 signaling pathway. While silenced C-erbB-2 expression and inactivated IGF-1 signaling pathway caused decreased mRNA and protein expressions of IGF-1, IGF-1R and Akt, decreased cell proliferation, migration and invasion, prolonged G0/G1 phase and shortened S phase, increased cell apoptosis, and inhibited tumor growth. By contrast, there were significant opposite trends after overexpressing C-erbB-2 expression and activating IGF-1 signaling pathway. These results indicate that up-regulation of C-erbB-2 can promote the growth and proliferation, invasion and migration, prolongation of S phase, and decrease cell apoptosis of EC cells, and down-regulation of C-erbB-2 can reverse this effect to exert a protective effect on EC. Meanwhile, silencing C-erbB-2 can inhibit the expression of proteins related to IGF-1 signaling pathway. Meanwhile, it is inferred that the inhibition of IGF-1 pathway can weaken the invasion, migration and proliferation of EC cells, and promote apoptosis, while IGF-1R pathway agonists can reverse the ability of promoting EC cell invasion, migration and proliferation, and inhibit apoptosis. More importantly, supplemented GF1 signaling pathway agonists can reverse the positive effects of C-erbB-2 silencing on cell proliferation, migration, invasion, cell cycle and apoptosis. Besides, animal model suggests that the overexpression of C-erbB-2 and activation of IGF-1 signaling pathway can promote rapid growth and metastasis of tumor cells, while low expression of C-erbB-2 and inhibited activation of IGF-1 signaling pathway can inhibit the growth and metastasis of EC cells.

Significantly, uncontrolled cell growth is a sign of the occurrence and development of malignant tumors [43]. MTT colorimetry is a non radioactive quantitative method for cell viability [44]. Apoptosis refers to the programmed cell death, which is a self-regulation mechanism to remove redundant, aging and damaged cells during development and in response to external stimulation, so as to maintain internal balance and normal physiological activities [45]. In addition, the invasion and destruction of extracellular matrix by tumor cells and their migration into the vascular system is an important link in the process of metastasis [46]. The invasion ability of tumor cells in vitro (Transwell assay for example) can reflect their metastatic potential [47]. Our study explored and elaborated innovatively the beneficial roles of silencing C-erbB-2 and inactivating IGF-1 signaling pathway in suppressing EC progression in vivo and in vitro. This study is the first to reveal that the effect of intervening C-erbB-2 expression on EC may be associated with the activation of IGF-1 signaling pathway to affecting the invasion and metastasis of EC cells. Besides, the growth, invasion and apoptosis of Eca-109 cells were observed comprehensively after suppressing the expression of C-erbB-2 in EC cells, which proved the significance of target gene expression silencing in the treatment of EC. Moreover, further verification in animals was carried out based on in vivo exploration.

## Conclusion

To sum up, there may be high expression of C-erbB-2 and activation of IGF-1 signaling pathway in EC. Silencing C-erbB-2 gene expression may inhibit the proliferation of EC cells, promote cell apoptosis and block cell cycle progression by inhibiting the activation of IGF-1 signaling pathway. Meanwhile, the beneficial effect of silencing C-erbB-2 gene expression can be reversed by activating IGF-1 signaling pathway. In future, we will emphasize on the specific mechanism of C-erbB-2 gene regulating the activation of IGF-1 signal pathway, and further explore the mechanism of C-erbB-2 in regulating biological behaviors of EC cell.

## Data Availability

Not applicable.

## Abbreviations

EC: Esophageal carcinoma
IGF-1: Insulin like growth factor 1
IGF-1R: Insulin like growth factor 1 receptor
RNAi: RNA interference
BSA: Bovine serum albumin
NC: Negative control
cDNA: Complementary DNA
